# High concentrations of morphine sensitize and activate mouse dorsal root ganglia via TRPV1 and TRPA1 receptors

**DOI:** 10.1186/1744-8069-5-17

**Published:** 2009-04-16

**Authors:** Alexander B Forster, Peter W Reeh, Karl Messlinger, Michael JM Fischer

**Affiliations:** 1 Institute of Physiology and Pathophysiology, University of Erlangen-Nürnberg, Universitätsstrasse 17, Erlangen, 91054 Germany; 2 Department of Pharmacology, University of Cambridge, Tennis Court Rd, Cambridge, CB2 1PD, UK

## Abstract

**Background:**

Morphine and its derivatives are key drugs in pain control. Despite its well-known analgesic properties morphine at high concentrations may be proalgesic. Particularly, short-lasting painful sensations have been reported upon dermal application of morphine. To study a possible involvement of TRP receptors in the pro-nociceptive effects of morphine (0.3 – 10 mM), two models of nociception were employed using C57BL/6 mice and genetically related TRPV1 and TRPA1 knockout animals, which were crossed and generated double knockouts. Hindpaw skin flaps were used to investigate the release of calcitonin gene-related peptide indicative of nociceptive activation.

**Results:**

Morphine induced release of calcitonin gene-related peptide and sensitized the release evoked by heat or the TRPA1 agonist acrolein. Morphine activated HEK293t cells transfected with TRPV1 or TRPA1. Activation of C57BL/6 mouse dorsal root ganglion neurons in culture was investigated with calcium imaging. Morphine induced a dose-dependent rise in intracellular calcium in neurons from wild-type animals. In neurons from TRPV1 and TRPA1 knockout animals activation by morphine was markedly reduced, in the TRPV1/A1 double knockout animals this morphine effect was abrogated. Naloxone induced an increase in calcium levels similar to morphine. The responses to both morphine and naloxone were sensitized by bradykinin.

**Conclusion:**

Nociceptor activation and sensitization by morphine is conveyed by TRPV1 and TRPA1.

## Background

After many thousands of years of therapeutic opium use, in 1806 the German pharmacist Sertuerner published the discovery of the main pharmacological compound of opium, which he later called morphine [1]. From this time morphine and its derivatives, which form the class of opioids, became the main therapeutic option for the treatment of moderate to severe pain. The analgesia as well as most side effects are caused by interaction with the opioid receptors, especially the mu-receptors.

Opioids have several commonly known side effects including addiction, sedation, constipation and nausea [2]. Besides their well-known antinociceptive actions, opioids can cause hyperalgesia by an unknown, opioid receptor-independent mechanism [3,4]. This present study was initiated by the clinical observation that in patients suffering from radiodermatitis, which could not be controlled by systemic morphine, high doses of a custom-made opioid gel applied topically to the skin can elicit severe burning pain for a few seconds (Clinical research group KFO130, Erlangen, Germany). The isotonic gel prepared by the university's pharmacy contains 2.67 mM (0.1%) morphine and has a neutral pH. Case reports have previously reported hyperalgesia after application of high doses of morphine [5,6]. At lower concentrations morphine only causes pruritus, which might be explained by mast cell degranulation [7].

The transient receptor potential channel TRPV1 (formerly VR1) receptor is found in small to medium diameter dorsal root, trigeminal and nodose ganglia primary afferent neurons which sense potentially damaging stimuli such as capsaicin, heat and low pH [8,9]. For TPRV1 more pungent activating compounds have been reported than for any other TRPV channel. Another TRP channel, TRPA1 (formerly ANKTM1) equips neurons with a sensitivity for mustard oil and many diverse pungent chemical stimuli [10]. Among these compounds are electrophilic compounds such as allyl isothiocyanate, acrolein, formalin, 15-deoxy-Δ12,14-prostaglandin J2, nitric oxide, hydrogen peroxide as well as the inflammatory mediator bradykinin [11]. TRPA1 and TRPV1 are the principal detectors for painful chemical stimuli. The aim of this study was to investigate whether the painful sensations evoked by morphine are mediated by these two receptors. Two models of nociceptive activation were employed using mice lacking TRPV1, TRPA1 or both these receptors.

## Results

### Morphine activates hTRPV1 and TRPA1 expressed in HEK293t

**Figure 1:**
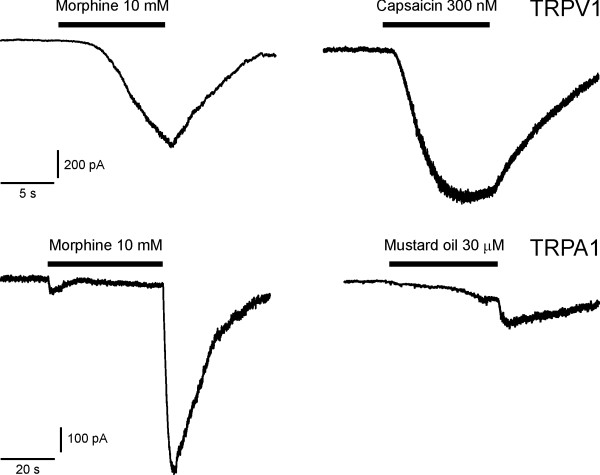
**Morphine activates TRPV1 and TRPA1 expressed in HEK cells**. HEK cells transfected with cDNA for hTRPV1 and mTRPA1 were exposed to 10 mM morphine. The upper traces show an inward current in a cell transfected by hTRPV1 and GFP, evoked by 10 mM morphine and by 300 nM capsaicin, both applied for a period of 10 s. The lower traces show an inward current in a cell transfected by mTRPA1 and GFP, evoked by 10 mM morphine and by 30 μM mustard oil, both applied for a period of 40 s. Cells were exposed to 1 μM m-3M3FBS before application of morphine and between applications.

In HEK293t cells transfected with hTRPV1 application of 10 mM morphine evoked an inward current, 300 μM capsaicin was applied as a control thereafter (n = 10, sample recording in Fig. 1). In HEK293t cells transfected with mTRPA1 10 mM morphine evoked inward currents that appeared to rapidly inactivate during continued superfusion (n = 10). However, upon offset and washout of morphine regularly large tail currents occurred that revealed the sustained activation of TRPA1 and a slow deactivation. Mustard oil (30 μM) was applied as a control thereafter.

### Morphine elicits calcium transients in DRG neurons

**Figure 2:**
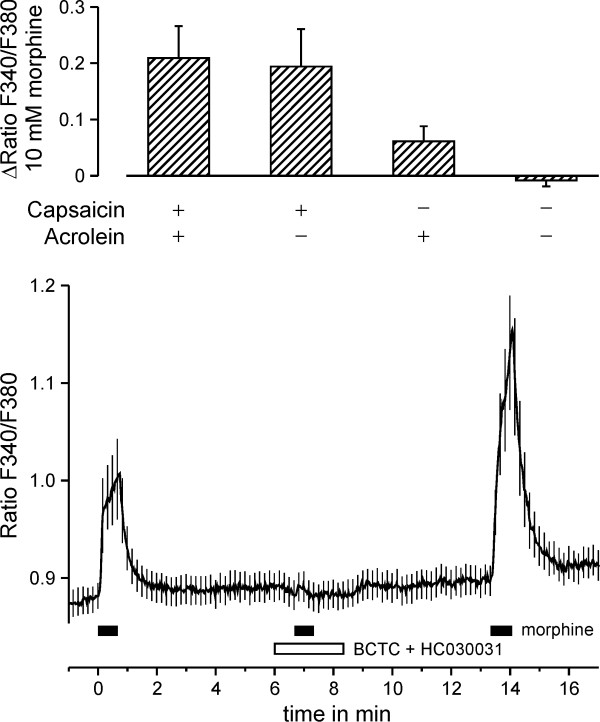
**Morphine activates DRG neurons responsive to capsaicin and acrolein**. Upper panel. Morphine, acrolein and capsaicin were applied to 106 wildtype DRG neurons. The average calcium increase evoked by morphine 10 mM is compared in neurons grouped by sensitivity to capsaicin and acrolein. Neurons responsive to capsaicin, acrolein or both showed a larger calcium signal response to morphine than neurons not responsive to both capsaicin and acrolein. Lower panel. Wildtype DRG neurons were exposed to three repetitive applications of 10 mM morphine (n = 62). In the presence of 10 μM BCTC and 50 μM HC030031, morphine evoked no significant increase in intracellular calcium.

A total of 106 dorsal root ganglion (DRG) neurons were exposed to 10 mM morphine for 40 s, 30 μM acrolein for 30 s, 1 μM capsaicin for 5 s and 60 mM potassium for 30 s with an interval of 240 s between applications. Response rates to morphine were 22%, to acrolein 45% and to capsaicin 58%. Morphine responses in neurons activated by capsaicin but not by acrolein and in neurons activated by acrolein but not capsaicin were larger than in neurons which did not respond to either capsaicin or acrolein (p = 0.024 and p = 0.027, t-test independent samples). Responses to morphine grouped by the sensitivity to capsaicin and acrolein are presented in Fig. 2a. Morphine (10 mM) was applied three times for 40 s. The second application of morphine in the presence of BCTC (10 μM) and HC030031 (50 μM) did not elicit a calcium increase (both p < 0.001 compared to the initial and the third morphine application, n = 62, Wilcoxon, Fig. 2b).

**Figure 3:**
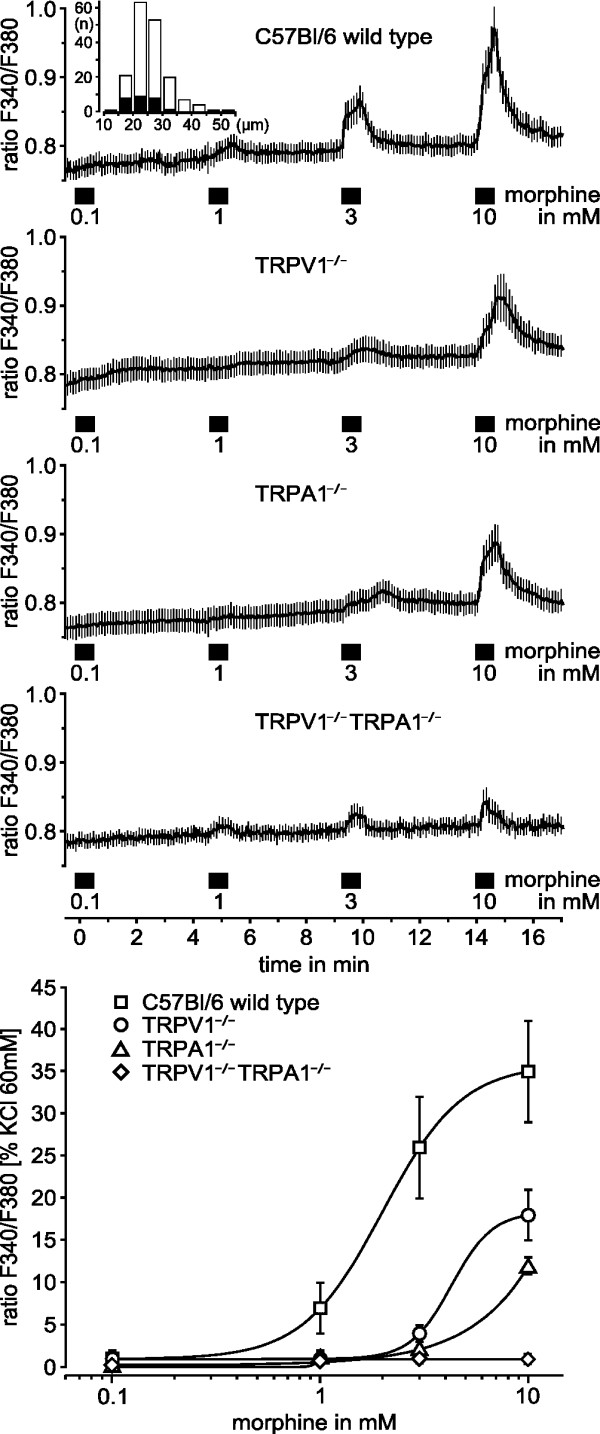
**Morphine dose-dependently activates DRG neurons via TRPA1 and TRPV1 receptors causing increases in intracellular calcium**. Morphine application periods lasting 40 s are indicated by bars. The upper four panels show the fluorescence ratios of the four tested genotypes, presented as mean ± SEM of all neurons (number of neurons, C57Bl/6: 134, TRPV1^-/-^: 112, TRPA1^-/-^: 125 and TRPV1^-/- ^TRPA1^-/-^: 135). All neurons responded to 60 mM KCl at the end of the experiment (not shown). The stacked histogram inset in the uppermost panel shows that preferentially small size DRG neurons respond to morphine (black bars, x: cell diameter in μm, y: number of cells). The bottom panel summarizes the dose-dependent activation. The integral of the fluorescence ratio during the application period was normalized to the response elicited by 60 mM KCl.

Increasing concentrations of morphine (0.1 – 10 mM) were consecutively applied for 40 s, separated by 240 s washout intervals. From C57BL/6 animals 134 DRG neurons were tested. Morphine at 3 mM and 10 mM evoked a concentration-dependent transient rise in intracellular calcium (ANOVA, F_(4,532) _= 16.9, p = 0.005 and p < 0.001, HSD post-hoc tests). The EC_50 _was 2.6 ± 0.04 mM (Fig. 3). Morphine at 10 mM reached 32% of the 60 mM potassium-induced calcium influx.

This protocol was repeated in neurons from TRPV1^-/- ^(n = 112), TRPA1^-/- ^(n = 125) as well as from TRPV1^-/- ^TRPA1^-/- ^animals (n = 135). Compared to neurons from C57BL/6 mice, the increases in calcium levels evoked by 10 mM morphine were smaller in animals lacking TRPV1 or TRPA1 and almost absent in double knockouts (ANOVA, F_(9,1485) _= 7.0; TRPV1^-/-^: 51%, p = 0.008; TRPA1^-/-^: 34%, p = 0.005; TRPV1^-/- ^TRPA1^-/-^: 2%, p < 0.001; % of AUC in C57BL/6, HSD post-hoc tests). Compared to baseline, the small morphine-induced increases of intracellular calcium were still significant in neurons from TRPV1^-/- ^(p < 0.001, ANOVA, F_(4,444) _= 13.0) and TRPA1^-/- ^(p < 0.001, ANOVA, F_(4,468) _= 24.6), but not so in neurons from TRPV1^-/- ^TRPA1^-/- ^animals (p = 0.10, ANOVA, F_(4,536) _= 2.0). Due to the limited solubility of morphine at pH 7.4, no higher concentrations than 10 mM could be tested. Neurons activated by morphine were 20.5 ± 0.9 μm in diameter, smaller than the average of 23.0 ± 0.5 μm (p = 0.041, U-test).

### Sensitization by bradykinin and effect of naloxone

**Figure 4:**
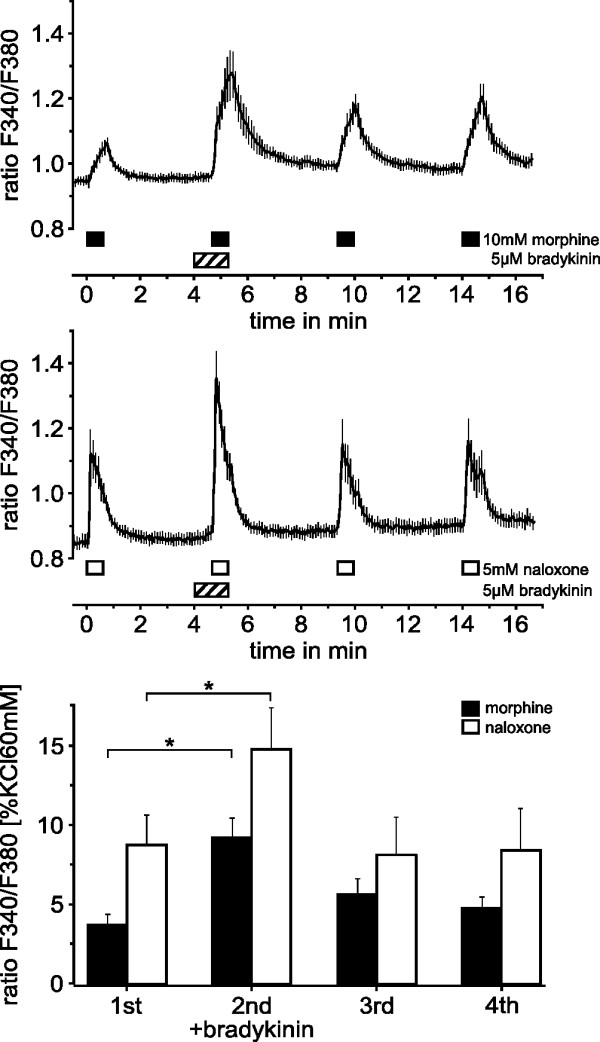
**Bradykinin sensitizes activation of neurons by morphine and naloxone**. Repetitive activation of DRG neurons elicited by 40 s applications of morphine and naloxone. Bradykinin reversibly sensitized calcium influx evoked by both morphine (n = 111) and naloxone (n = 91) but did not evoke a response when applied alone 40 s prior to morphine or naloxone. Bars indicate application periods; data are presented as mean ± SEM. The lower panel summarizes the calcium increases normalized to individual responses to 60 mM KCl. *p < 0.05

The inflammatory mediator bradykinin is known to activate nociceptive neurons in higher concentrations but in lower concentrations to sensitize TRPV1 and TRPA1. To determine whether calcium increases evoked by 10 mM morphine are increased by bradykinin at a concentration below activation, we applied the second of four consecutive morphine applications in the presence of 5 μM bradykinin (Fig. 4). Bradykinin was applied 40 seconds before the morphine stimulus and did not elicit a calcium response (n = 111). Among the 22.5% of DRG neurons responding to morphine, bradykinin increased the morphine-stimulated calcium influx to 249% (ANOVA, F_(3,330) _= 5.1, p= 0.002, HSD post-hoc test), the subsequent two morphine responses were not different compared to the first one (p = 0.33 and p = 0.87, HSD post-hoc tests). This protocol was repeated with morphine replaced by naloxone, which is structurally similar but has no intrinsic activity on opioid receptors. The activation by naloxone was also concentration-dependent (n = 10, data not shown). Bradykinin reversibly increased the naloxone-stimulated calcium increase to 165% of the first response (ANOVA, F_(3,66) _= 5.0, p = 0.025, HSD-post hoc test).

### Morphine-induced CGRP release from hindpaw skin

**Figure 5:**
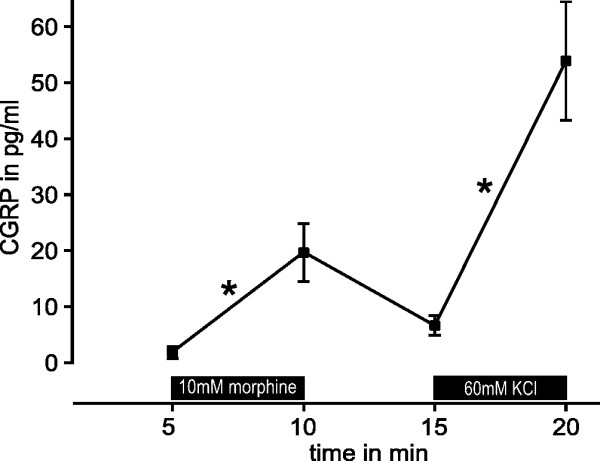
**Morphine elicits release of CGRP**. Morphine (10 mM) applied for 5 minutes reversibly stimulates the release of CGRP from the isolated hindpaw skin of mice (n = 8). Synthetic interstitial fluid containing 60 mM KCl was applied for comparison. *p < 0.05

In order to reappraise these findings in intact innervated tissue, we investigated the stimulated CGRP release from nerve endings in isolated hind paw skin of C57BL/6 mice. In a first series of experiments (n = 8) we assessed the response to application of 10 mM morphine diluted in SIF (Fig. 5). The basal CGRP concentration in the first incubation period (SIF) was 2 ± 1 pg/ml. When the skin flaps were transferred to the test tube containing 10 mM morphine, the CGRP release increased to 20 ± 5 pg/ml (p = 0.012, Wilcoxon). Final exposure to 60 mM KCl resulted in a CGRP release of 54 ± 11 pg/ml.

### Morphine sensitizes CGRP release induced by acrolein and heat

**Figure 6:**
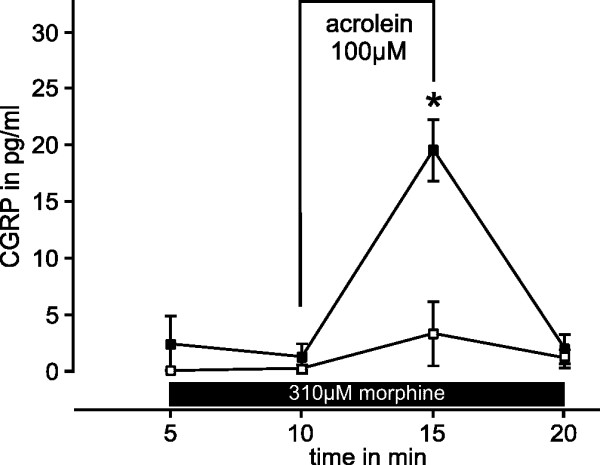
**Morphine sensitizes CGRP-release stimulated by acrolein**. Release of CGRP from isolated hindpaw skin of mice was stimulated by 100 μM acrolein applied for 5 min (open symbols). The contralateral hindpaw skin of every animal was exposed to acrolein in the presence of 310 μM morphine (closed symbols) which sensitized the release of CGRP evoked by acrolein (n = 6). *p < 0.05

**Figure 7:**
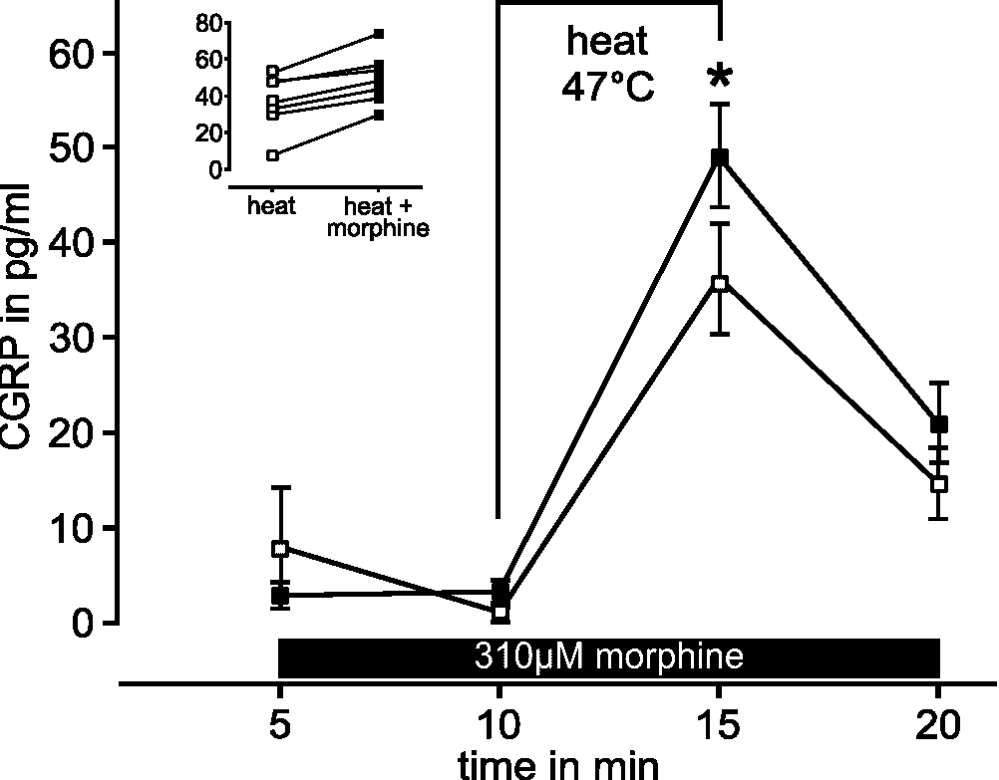
**Morphine sensitizes CGRP-release stimulated by noxious heat**. Release of CGRP from isolated hindpaw skin of mice stimulated by noxious heat of 47°C applied for 5 min (open symbols). The contralateral hindpaw skin flap of every animal was exposed to 47°C in the presence of 310 μM morphine (closed symbols) which slightly facilitated the release of CGRP stimulated by heat (n = 7). The inset shows the results with and without morphine for every animal. *p < 0.05.

A separate series of experiments was performed to test the hypothesis that morphine concentrations without a direct effect on CGRP release may sensitize cutaneous afferents to noxious stimuli activating TRPV1 or TRPA1. CGRP release in the presence of 310 μM morphine was not different from the basal release (Fig. 6). The selective agonist acrolein 100 μM was used to activate TRPA1 at the lower end of the concentration-response relationship; in presence of 310 μM morphine acrolein caused markedly greater increases in CGRP release (p = 0.028, n = 6, Wilcoxon). A similar series of experiments was performed for noxious heat stimulation using SIF at 47°C; heat-induced cutaneous CGRP release depends partly on TRPV1 as a transducer. The exposure to 310 μM morphine caused no rise in CGRP release (Fig. 7), but slightly sensitized the release evoked by heat stimulation (p = 0.018, n = 7, Wilcoxon, compared to 47°C on the control side).

## Discussion

Morphine and naloxone at high concentrations were found to activate cultured primary afferent neurons in a concentration-dependent manner demonstrated by inward currents and calcium transients. TRPV1 and TRPA1 but not opioid receptors conveyed this activation. The inflammatory mediator bradykinin increased the neuronal sensitivity to morphine and naloxone. Morphine concentrations below the activation threshold sensitized TRPV1 and TRPA1 in native cutaneous nerve endings releasing CGRP.

### Clinical observation

The present study was initiated by the observation of painful sensations caused by topical application of a 2.67 mM morphine gel to inflamed skin following radiation therapy, as reported in the interdisciplinary pain research group (KFO 130). In these patients, the topical morphine formulation (prepared by the pharmacy unit of the university) was preferred because systemic opiates could not be applied systemically due to unwanted side effects. The painful sensations were reported to last only for a few seconds but exceeded seven on a ten-point rating scale. The time course of the pain was similar to that reported after injection of lidocaine, which has recently been shown to activate nociceptive afferents via TRPV1 and TRPA1 receptors before sodium channel blockade leads to local anesthesia [12]. Similarly for morphine, an inhibition of sodium channels has been described [13-15], masking by conduction block the sustained activation. Sodium channel block also occurs with naloxone [16], shedding doubts on alleged naloxone reversal of peripheral opioid effects.

The radiation dermatitis involves a tailored therapy; topical application of morphine is not the primary choice but rather an uncommon option. This might explain why systematic studies are not available. Common symptomatic therapy of radiation dermatitis is based on topical wound care predominantly involving moisturizers. There is no uniequivocal evidence for a causal therapy, including the topical application of ascorbic acid, corticosteroids or calcineurin inhibitors; opioids have not been investigated so far [17].

### Receptors mediating opioid effects

There is evidence for peripheral opioid receptor effects [18-20]. The expression profile of opioid receptors on primary afferent neurons corresponds to the nociceptor population [21,22]. Peripheral application of opioid receptor agonists (below 100 μM) or antagonists demonstrated their local efficacy but the relative contribution of peripheral opioid receptors after systemic opioid administration is still unclear [23].

In animal experiments involving peripheral opioid injections or topical morphine applications, pain-related behavior has not been reported [24-26]. However, it seems likely that in these studies morphine concentrations at the nerve endings were below the activation threshold of nociceptors. A behavioural correlate for the activation of TRPV1 and TRPA1 by morphine injection is not easily detected since the immediate pain of skin puncture is closely followed by the nerve conduction block. The only previously reported application of a high concentration of morphine in a well-controlled setup produced nociceptor activation, as observed in the present study. In this preparation, the superior spermatic nerve of dogs in vitro, morphine applied by superfusion at 1 – 310 μM generated action potentials in slowly conducting afferents; the activation was concentration-dependent and the combination of bradykinin and morphine resulted in sensitization [27]. In the present study the applied concentrations were higher, and 310 μM morphine caused sensitization but no overt activation. A higher expression of TRPA1 and TRPV1 in visceral afferents [28,29] might contribute to the lower concentration of morphine required for activation of testicular nociceptors.

For the reported painful sensations in patients or the activation of primary afferents, a G-protein coupled opioid receptor effect seems unlikely in view of the action of the opioid antagonist naloxone. Since TRPV1 and TRPA1 are the principal detectors for painful chemical compounds, they were the primary targets of the investigation. Considering pharmacological results and activation of neurons from knockout animals, both TRP receptors contributed approximately equal to the activation of DRG neurons caused by morphine. Only the knockout of both receptors eliminated the responsiveness.

The observed morphine-induced increase in calcium levels is only a fraction of that elicited by KCl. This might explain why a lower percentage of neurons was activated by morphine in comparison to acrolein and capsaicin. Inhibition of calcium conductance by morphine is a possible explanation. In fact, morphine and the mu-agonist DAGO (Tyr-D-Ala-Gly-MePhe-Gly-ol) reduce the conductivity of voltage gated calcium channels [30,31], which may be activated secondary to neuronal depolarization via TRPV1 and TRPA1. For TRPA1, both morphine and mustard oil seem to inhibit ion flux through TRPA1, a tail current was observed when the application was stopped. The apparent desensitization is likely due to an additional pore blocking effect that morphine at high concentration may exert on TRPA1. Mustard oil, the index agonist of TRPA1, also induced, yet smaller, tail currents followed by slow deactivation. Together with the very slow activation this may suggest that also mustard oil exerts a double action on TRPA1, activation of the receptor-channel and partial pore block.

### Stimulated CGRP release

The neuropeptide calcitonin gene related peptide CGRP is expressed by a large subset of predominantly nociceptive primary afferent neurons and released upon activation of these neurons. The measurement of released CGRP is therefore widely used as an index for the activation of nociceptors. CGRP is expressed in a large proportion of neurons positive for TRPV1 or TRPA1, therefore the release of CGRP is a favorable readout for the activation of these neurons [32,33]. High concentrations of morphine reversibly stimulated the release of CGRP from the isolated skin. Furthermore, concentrations below the threshold of activation sensitized the CGRP release evoked by noxious heat and acrolein, activators of TRPV1 and TRPA1 receptors, respectively.

In inflamed skin inflammatory mediators and their receptors are frequently overexpressed [34,35], among them particularly bradykinin and its receptors [36]. Bradykinin sensitizes TRPV1 [37] and TRPA1 [38]. Therefore it was not surprising that morphine responses were sensitized by bradykinin. Sensitization to morphine by bradykinin and a reduced barrier function may explain the particular painfulness of the treated skin areas in radiodermatitis patients.

## Conclusion

Morphine, at doses rarely used for pain therapy, is a noxious stimulus activating both TRPV1 and TRPA1 receptors. Morphine-induced calcium increase, indicative for nociceptive activation, was reduced in DRG neurons from TRPV1 and TRPA1 knockout animals. Moreover, TRPV1/A1 double knockout mice were used for the first time to demonstrate by the lack of activation that both receptors together fully account for the nociceptive sensitivity to morphine. We suggest that also the sensitivity for several other algogenic compounds that still elicit pain and activate neurons in the single knockouts will be lost in these double knockout animals.

## Methods

### Animals

Animal care and treatment were in accordance with the guidelines of the International Association for the Study of Pain [39]. Adult C57Bl/6, TRPV1^-/- ^and TRPA1^-/- ^as well as TRPV1^-/-^TRPA1^-/- ^double knockout mice were used. Breeding pairs of TRPV1 and TRPA1 knockout mice were obtained from Dr. John Davis [40] and Dr. David Corey [41] and backcrossed to C57BL/6. Double knockout animals were generated in our animal facility by cross-mating knockouts of both strains. All animals were genotyped by the previously reported primers.

### Cell culture

Animals were killed in pure CO_2 _atmosphere. Dorsal root ganglia of all lumbar and the first two thoracic segments of the spinal column were excised and transferred in Dulbecco's modified Eagle's medium (DMEM, Invitrogen, Carlsbad, USA) solution containing 50 μg/ml gentamicin (Sigma). The ganglia were treated with 1 mg/ml collagenase and 0.1 mg/ml protease for 30 minutes (both from Sigma) in TNB 100 solution supplemented by TNB 100 lipid-protein-complex, 100 μg/ml streptomycin and penicillin (all from Biochrom, Berlin, Germany) and 200 μg/ml glutamine (Invitrogen, Carlsbad, USA) for 20 minutes at 37°C. After three wash steps, the suspension was triturated in TNB solution using a fire-polished silicone-coated Pasteur pipette. The cells were plated on poly-D-lysine-coated (Sigma) cover slips and cultured in TNB medium overnight at 37°C in a humidified 5% CO_2 _atmosphere.

### Patch Clamp Recordings

HEK293t cells were transfected with plasmids of hTRPV1 (John Davis) or mTRPA1 (Ardem Patapoutian) along with GFP as reporter plasmid using Polyfect (Qiagen, Hilden, Germany) according to the manufacturer's protocol. After incubation for one day, the cells were replated in 35 mm culture dishes and used for experiments within 1–2 days. GFP-expressing cells were identified by fluorescence.

Recording electrodes were pulled from borosilicate glass tubes (GB150T-8P, Science Products, Hofheim, Germany) to give a resistance of 3.5 – 5.0 MΩ (P97, Sutter, Novato, CA). The external solution contained (in mM) 140 NaCl, 4 KCl, 1.8 CaCl_2_, 1 MgCl_2_, 10 HEPES, 4 glucose, adjusted to pH 7.4. The internal solution contained (in mM) 140 KCl, 1.6 MgCl_2_, 2 EGTA and 10 HEPES and was adjusted to pH 7.4. Recordings were performed at room temperature and cells were held at -60 mV. Membrane currents were acquired with an Axopatch 200B amplifier and pClamp 10 software (Molecular Devices, Sunnyvale, CA). Solutions were applied with a RSC-100 gravity-driven solution changer (Bio-Logic, Claix, France). All cells were first exposed to morphine, then to capsaicin or mustard oil. TRPA1 was sensitized by PLC activator m-3M3FBS (1 μM, Sigma).

### Calcium imaging

Cells were stained by 5 μM fura-2 AM and 0.02% pluronic F-127 (both from Invitrogen) dissolved in the TNB medium for about 30 min in the incubator, followed by a short wash-out period to allow fura-2 AM ester hydrolysis. The cover slips were placed in a custom-made chamber and mounted on a Zeiss Axiovert inverse microscope with a 40× NeoFluar objective. Cells were continuously superfused throughout the experiment with extracellular fluid (in mM: NaCl 145, KCl 5, CaCl_2 _1.25, MgCl_2 _1, Glucose 10, Hepes 10) at a flow rate of approximately 0.3 ml/min at room temperature. Cells were illuminated with a 75W xenon arc lamp and a monochromator alternating between 340 and 380 nm of wavelength (Photon Technology International, New Jersey, USA). Images were acquired at 1 Hz with 200 μs exposure time using a CCD camera, controlled by Image Master software (PTI, Birmingham, USA). Fluorescence ratios were computed for regions of interest adapted to the neurons. All experimental protocols were pre-programmed using a custom-made software that controls the valves of a 7-channel gravity-driven common-outlet superfusion system [42].

### CGRP release

C57/Bl6 mice of either sex with an average weight of 20 g (range 12–27 g) were used. The skin of both hind paws distal to the knee was subcutaneously excised. The skin flaps with an average weight of 105 mg (range 57–135 mg, n = 36) were fixed to acrylic rods by surgical threads with the corium exposed. During this procedure the skin flaps were constantly immersed in synthetic interstitial fluid (SIF, in mM: NaCl 107.8, KCl 3.5, NaHCO_3 _26.2, NaH_2_PO_4 _1.7, Na-gluconate 9.6, sucrose 7.6, glucose 5.6, CaCl_2 _1.5 and MgSO_4 _0.7 [43] equilibrated with carbogen (pH 7.4). The fixed skin flaps were placed in test tubes and mounted in a shaking bath of 32°C. After washing for 30 minutes, each experiment was composed of four consecutive 5 min incubation periods in test tubes filled with 0.8 ml SIF. In the first two periods basal CGRP release was determined, during the third period the preparation was chemically or thermally stimulated, the fourth period was for the post-stimulation control. One separate protocol provided two stimulations in incubation periods two and four. The contralateral hind paw skin of each animal was used as a matched-pairs control without conditioning chemicals.

### Enzyme-immuno assay for CGRP

Immediately after the incubation, 100 μl of the solution were processed using a commercial CGRP enzyme immune assay kit (SPIbio, France). The details of this method were described previously [44]. The antibodies used are directed against human α/β-CGRP but are 100% cross-reactive against mouse and rat CGRP. The detection level is about 2 pg/ml. Samples of SIF and all stimulation solutions measured on the same enzyme immune assay 96-well plate served as controls.

### Data analysis

The calcium influx elicited by a stimulus was quantified by evaluating the area under the curve (AUC) of the application period. Absolute increases in calcium concentrations were calculated based on R_min _and R_max _[45]. Compared to the foregoing reference period a mean increase of the intracellular calcium concentration of at least 15 nM throughout the application period was considered as activation. At the end of all protocols a 10 s stimulus of KCl 60 mM was applied as control and normalization reference. Cell diameter was calculated as the mean of two orthogonal axes. Repeated measurements and independent groups in calcium imaging experiments were compared by ANOVA and HSD post-hoc test. CGRP release experiments were performed using both hindpaw skin flaps of the same animal and compared using the Wilcoxon matched pairs test. Statistical analysis was performed using Statistica 7 (Tulsa, OK, USA), sigmoidal dose-response curves were fitted by Origin 7.5 (Northhampton, MA, USA). Data are presented as mean ± SEM; p < 0.05 was considered significant.

## Abbreviations

CGRP: calcitonin gene-related peptide; DRG: dorsal root ganglion; TRPV1: transient receptor potential cation channel, subfamily V, member 1; TRPA1: transient receptor potential cation channel, subfamily A, member 1; SIF: synthetic interstitial fluid

## Competing interests

The authors declare that they have no competing interests.

## Authors' contributions

ABF carried out the experiments and performed the statistical analysis. KM and PWR helped in experimental design and to draft the manuscript. MJMF designed the study, supervised all aspects and drafted the manuscript. All authors read and approved the final manuscript.
